# Sleep disturbances among women in a Subarctic region: a nationwide study

**DOI:** 10.1093/sleep/zsac100

**Published:** 2022-05-09

**Authors:** Anna Bára Unnarsdóttir, Arna Hauksdóttir, Thor Aspelund, Vigdís Gunnarsdóttir, Gunnar Tómasson, Jóhanna Jakobsdóttir, Unnur Anna Valdimarsdóttir, Edda Bjork Thordardottir

**Affiliations:** Centre of Public Health Sciences, Faculty of Medicine, School of Health Sciences, University of Iceland, Reykjavik, Iceland; Centre of Public Health Sciences, Faculty of Medicine, School of Health Sciences, University of Iceland, Reykjavik, Iceland; Centre of Public Health Sciences, Faculty of Medicine, School of Health Sciences, University of Iceland, Reykjavik, Iceland; Centre of Public Health Sciences, Faculty of Medicine, School of Health Sciences, University of Iceland, Reykjavik, Iceland; Centre of Public Health Sciences, Faculty of Medicine, School of Health Sciences, University of Iceland, Reykjavik, Iceland; Department of Rheumatology, University Hospital, Reykjavik, Iceland; Centre of Public Health Sciences, Faculty of Medicine, School of Health Sciences, University of Iceland, Reykjavik, Iceland; Centre of Public Health Sciences, Faculty of Medicine, School of Health Sciences, University of Iceland, Reykjavik, Iceland; Department of Medical Epidemiology and Biostatistics, Karolinska Institutet, Stockholm, Sweden; Department of Epidemiology, Harvard T.H. Chan School of Public Health, Boston, MA, USA; Centre of Public Health Sciences, Faculty of Medicine, School of Health Sciences, University of Iceland, Reykjavik, Iceland

**Keywords:** sleep, PSQI, Subarctic, Iceland, women, population, seasons

## Abstract

**Study Objectives:**

To date, few studies have assessed sleep problems among women residing in Subarctic regions. Therefore, the aim of this large-scale population-based study was to assess the prevalence of severe sleep problems and associated factors among Icelandic women, living at 63–66°N.

**Methods:**

Participants were 29 681 women (18–69 years old) who took part in the Icelandic Stress-And-Gene-Analysis study in 2018–2019. Background information, health-related behavior, and mental health symptoms were assessed with an online questionnaire. The Pittsburgh Sleep Quality Index (PSQI) was used to assess severe sleep problems during the past month. Adjusting for age, marital status, number of children, education, personal income, work schedule, region, and response period, we used modified Poisson log-linear models to obtain prevalence ratios (PRs) with 95% confidence intervals (CIs).

**Results:**

Overall, 24.2% of women reported severe sleep problems (PSQI >10). Women responding in the winter presented with an overall higher prevalence of severe sleep problems, compared to those responding in the summer (PR 1.21; 95% CI, 1.15 to 1.28). Severe sleep problems were more prevalent among young and late-midlife women, those who were single, had children, socio-economic challenges, worked shifts, and flexible hours. Furthermore, obesity, suboptimal health behaviors, excessive screen time, and mental health problems were associated with severe sleep problems.

**Conclusion:**

Severe sleep problems are more common among women in Subarctic regions than elsewhere, particularly during winter. These findings motivate the development of preventive strategies and interventions for women in the Subarctic who suffer from sleep problems.

**Statement of Significance**

Limited studies have assessed sleep problems among women living in Subarctic regions, areas with extreme variation in seasonal light duration, with long dark periods during winter and constant daylight during summer. Our population-based study of 29 681 women in Iceland indicates that severe sleep problems are more prevalent among women responding during the winter months when daylight is limited. We also found socioeconomic challenges, shift work, and suboptimal health behaviors to be associated with higher prevalence of sleep problems among women in the Subarctic region of Iceland with high-social welfare. These results are valuable for identifying women with higher likelihood of sleep disruptions and therefore would benefit from targeted prevention, in the Subarctic.

## Introduction

Sleep is essential for human’s overall well-being and general health [1], with most healthy adults needing at least 7 h of sleep each night to function properly and to avoid sleep deprivation [2]. Studies on sleep quality include both quantitative aspects, such as sleep length, sleep latency, and number of awakenings during the night, as well as qualitative aspects, such as feeling of being energetic and restored upon awakening [3]. Poor sleep quality has been associated with adverse long-term health consequences [4, 5], as well as higher risk of all-cause mortality [6].

Previous population-based studies indicate that 27%–52% of women experience sleep problems (Pittsburgh Sleep Quality Index [PSQI] > 5) [7–12] and 11%–28% severe sleep problems (PSQI > 10) [8, 13, 14]. Evidence suggests that insomnia, defined as having difficulties falling asleep or staying asleep [15], is more common among women in Subarctic regions (the Northern hemisphere), with 8%–16% of women suffering from insomnia in these regions [16–18], compared to 7%–12% women globally [19]. Winters in the Subarctic regions consist of dark periods while summers are marked by constant daylight [20]. It has been suggested that these light exposure patterns cause abnormalities of circadian rhythms and disturbed sleep [21–23]. Indeed, individuals who live near the Subarctic experience greater seasonal changes in insomnia and fatigue, compared to those who live near the equator [21]. However, few population-based studies have assessed sleep problems among Subarctic women living with extreme variation in seasonal light duration.

Previous results indicate that higher age [17], being in a relationship [24, 25], and having children [26, 27] are associated with poor sleep quality. Furthermore, previous studies have found low socio-economic status (SES) [7, 8, 28, 29] and shift work [30, 31] to be associated with sleep problems. In addition, high body max index (BMI) [8, 32, 33] and suboptimal health behaviors, such as cigarette smoking [8, 9, 34, 35], excessive alcohol consumption [36, 37], and prolonged screen time [38–40] have been associated with sleep problems.

Sleep problems are also highly comorbid with many major mental disorders. It has been suggested that sleep problems are a contributory factor in the occurrence of disorders such as anxiety and depression [41]. However, few studies have assessed the degree to which these situational and health-related factors impact sleep among those residing in the Subarctic region.

There are some indications that sleep deprivation may be a problem in Iceland, with a recent report finding 24% of Icelandic women to sleep on average <6 h per night [42]. Dispensing of hypnotics and sedatives, used in the treatment for insomnia, are also considerably higher in Iceland than in other Nordic countries [43].

To the best of our knowledge, no population-based study has to date assessed the prevalence of sleep problems among women residing in these Subarctic regions and associated factors. Epidemiological studies with representative general population-based samples using validated measurements are needed. Therefore, the aim of the current study was to leverage a population-based cohort of Icelandic women to investigate the prevalence of sleep problems with regard to season and latitude of residency. Furthermore, to assess the association of severe sleep problems and demographic- and socioeconomic characteristics, as well as health behaviors.

## Methods

### Study population

Participants were women who took part in the baseline assessment of the population-based SAGA (Stress-And-Gene-Analysis) cohort. All Icelandic speaking women, 18–69 years of age, residing in Iceland (*N =* 104 197 women) were actively recruited in the study. A total of 30 403 women (approximately 30% of eligible women in Iceland) participated in the study. The study population is representative of the total female population in terms of age, education, income, and geographic residence [44]. Participants with missing item(s) on the PSQI were excluded (*n* = 722), which resulted in a study population of 29 681 women (see flowchart in Supplementary Figure S1).

### Procedure

Women were invited to participate in the study through a phone text message or via mail. Participation included answering electronically an extensive internet-based questionnaire on mental and physical morbidities, including sleep disturbances. Data collection took place between March 2018 and July 2019.

### Measures

#### Background information.

Demographic information was collected for age (continuous, in years; categorized as: 18–29, 30–39, 40–49, 50–59, 60–69), marital status (married/in a relationship, single/divorced/widowed), number of children (continuous; 0, 1–2, 3–4, ≥5), education (primary, secondary, tertiary level A, tertiary level B), personal monthly income (<1200 Euro [EUR], 1201–2400 EUR, 2401–4000 EUR, 4001–5600 EUR, 5601–8000 EUR, >8001 EUR; conversion rates according to the Central Bank of Iceland, February 1, 2018) [45], employment status (active: working/studying/parental leave, inactive: on disability/sick leave/unemployed/retired), work schedule (fixed, flexible, shift work, unemployed), region (Reykjavik capital area, East Iceland, North Iceland, South Iceland, Southern Peninsula, Westfjords, West Iceland, living abroad), and response period (summer [June, July, August, September], fall [October, November], winter [December, January, February, March], spring [April, May]; classified according to the Icelandic Meteorological Office).

#### Health-related behavior and well-being.

Participants answered questions related to their general health, that is, BMI (continuous; categorized as: underweight [<18.5], normal weight [18.5–24.9], overweight [25.0–29.9], obese [≥30.0]), smoking (never, previous smoker, non-daily smoker, daily smoker; question based on the Fagerström Test for Nicotine Dependence) [46], alcohol consumption in the past year (i.e. six or more drinks on one occasion [never, less than once a month, monthly, once or more a week; based on an item from the Alcohol Use Disorders Identification Test]) [47], daily screen time during leisure time for the last week (i.e. in front of television, smartphone, and/or computer [<3 h, 3–5 h, 5–7 h, >7 h]). The Patient Health Questionnaire 9-item (PHQ-9) [48] was used to assess depressive symptoms in the past 2 weeks. Items were scored from 0 to 3 for symptom frequency. Standard cut-off scores were used: ≤9 indicating no or mild symptoms; 10–14 moderate symptoms; and **≥**15 moderately severe/severe symptoms [49]. The General Anxiety Disorder 7-item (GAD-7) [50] questionnaire was used to assess anxiety symptoms in the past 2 weeks. Items were scored on a four-point interval scale ranging from 0 to 3. Symptoms were classified as none/mild (score ≤ 9); moderate (score 10–14); and severe (score ≥15) [49].

#### Sleep quality.

The PSQI [3], a 19-item self-report scale, was used to assess sleep problems over a one-month time period. The items were scored on a four-point interval scale ranging from 0 to 3. The items generate seven components’ scores, including subjective sleep quality, sleep latency, sleep duration, habitual sleep efficiency, sleep disturbances, use of sleeping medication, and daytime dysfunction. The PSQI global score ranges from 0 to 21, with a lower score indicating better sleep quality, and a score above five indicating clinically significant sleep problems in at least two components or moderate difficulties in more than three components. A total PSQI score of >10 was used as a cut-off for severe sleep problems, in line with previous studies [8]. When analyzing the PSQI components separately, scores ranged from 0 (better) to 3 (worse) and a cut-off score of two was used to indicate symptomology, except for “use of sleep medication,” which was classified as present or absent (cut-off score > 1) and for “sleep length,” which was classified as sleeping more or less than seven hours (cut-off score > 1). The psychometric properties of the PSQI are adequate, particularly with regard to diagnostic sensitivity (89.6%) and specificity (86.5%) for insomnia [3]. The psychometric properties of the Icelandic version of the scale have demonstrated good internal consistency (i.e. Cronbach’s α = .82) [51].

### Statistics

Descriptive statistics were used to describe the distribution of demographic characteristics, residency, BMI, health-related behavior, well-being, and the seven PSQI components scores of the respondents. We used multiple binary logistic regression model to estimate the prevalence of severe sleep problems. The association of individual variables with severe sleep problems were presented as prevalence ratios (PRs) with 95% confidence intervals (CIs) by utilizing the modified Poisson model with the sandwich variance estimator [52, 53].

We divided the predictors into situational factors and factors related to health-behavior and well-being. A diagram explaining the relationship between the variables is included in the supplement (Supplementary Figure S2). We used multivariable adjustment for the association between situational factors and sleep (i.e. adjustments were made for age [using 10-year intervals], marital status, number of children, education, personal income, work schedule, region, and response period). The variables significantly associated with sleep were used as an adjustment when analyzing the association between severe sleep problems and behavioral factors and severe sleep problems and well-being (i.e. depression and anxiety).

Multiple imputation was used to replace missing values with *m* = 20 rounds of imputations and 20 iterations, using predictive mean matching [54]. The method of Benjamini-Hochberg for false discovery rate was used to correct for multiple comparison. We used a Wald test to test whether there was an effect modification by age groups. Cronbach’s α was estimated to determine internal consistency between the PSQI components. The statistical analyses were done using R (version 4.1.1).

## Results

### Study cohort

Data from 29 681 women contributed to this analysis. Characteristics of study participants are summarized in Table 1. The mean age of participants was 43.5 years (±13.7). The majority of the women were married or in a relationship (75.7%), had one or two children (39.3%), a university education (54.0%), 2401–4000 EUR monthly income (31.1%), were active in the labor market (85.2%), had a fixed work schedule (54.1%), and lived in the Reykjavik capital area (66.6%). In addition, 63.2% were overweight or obese, 15.5% were current smokers, and 13.9% binge drink at least once a month. The majority of women (53.0%) spent more than 3 hours every day on leisure-based screen time. Approximately one-third (29.1%) of women had moderate or severe depressive symptoms in the past 2 weeks and 22.6% had moderate or severe anxiety symptoms. Approximately half of the women answered the questionnaire during wintertime (Table 1).

**Table 1.** Characteristics of study participants*

	Overall (*n* = 29 681)	PSQI ≤ 10 (*n* = 22 484)	PSQI >10 (*n* = 7197)
Age			
Mean (*SD*)	43.5 (13.7)	43.4 (13.6)	44.1 (13.9)
Age range			
18–29	5887 (19.8%)	4486 (20.0%)	1401 (19.5%)
30–39	6131 (20.7%)	4744 (21.1%)	1387 (19.3%)
40–49	6554 (22.1%)	5035 (22.4%)	1519 (21.1%)
50–59	6671 (22.5%)	4948 (22.0%)	1723 (23.9%)
60–69	4438 (15.0%)	3271 (14.4%)	1167 (16.2%)
Marital status			
Married or in a relationship	22 359 (75.7%)	17 485 (78.1%)	4874 (68.3%)
Single, divorced, or widowed	7159 (24.3%)	4892 (21.9%)	2267 (31.7%)
Unknown	163	107	56
No. of children			
Mean (*SD*)	2.1 (1.4)	2.1 (1.4)	2.2 (1.5)
0	5230 (19.5%)	3999 (19.7%)	1231 (19.1%)
1–2	10 537 (39.3%)	8077 (39.7%)	2460 (38.1%)
3–4	9940 (37.1%)	7538 (37.1%)	2402 (37.2%)
≥5	1083 (4.0%)	725 (3.6%)	358 (5.5%)
Unknown	2891	2145	746
Education			
Tertiary level B	6578 (22.3%)	5572 (24.9%)	1006 (14.1%)
Tertiary level A	9378 (31.7%)	7466 (33.3%)	1912 (26.8%)
Secondary	9231 (31.2%)	6689 (29.8%)	2542 (35.6%)
Primary	4369 (14.8%)	2684 (12.0%)	1685 (23.6%)
Unknown	125	73	52
Personal income			
>8001 EUR	1032 (3.6%)	887 (4.1%)	145 (2.1%)
5601–8000 EUR	2901 (10.2%)	2461 (11.4%)	440 (6.4%)
4001–5600 EUR	6780 (23.8%)	5650 (26.2%)	1130 (16.4%)
2401–4000 EUR	8873 (31.1%)	6802 (31.5%)	2071 (30.0%)
1201–2400 EUR	6854 (24.0%)	4301 (19.9%)	2553 (36.9%)
<1200 EUR	2068 (7.3%)	1497 (6.9%)	571 (8.3%)
Unknown	*1173*	*886*	*287*
Employment status^†^			
Active	25 105 (85.2%)	20 140 (90.1%)	4965 (69.8%)
Inactive	4369 (14.8%)	2219 (9.9%)	2150 (30.2%)
Unknown	*207*	*125*	*82*
Work schedule			
Fixed	15 958 (54.1%)	13 255 (59.3%)	2703 (38.0%)
Flexible	3154 (10.7%)	2471 (11.1%)	683 (9.6%)
Shift work	2809 (9.5%)	2042 (9.1%)	767 (10.8%)
Unemployed	7553 (25.6%)	4591 (20.5%)	2962 (41.6%)
Unknown	*207*	*125*	*82*
Residence			
Reykjavik capital area	19 735 (66.6%)	15 284 (68.1%)	4451 (62.0%)
East Iceland	757 (2.6%)	570 (2.5%)	187 (2.6%)
North Iceland	3087 (10.4%)	2220 (9.9%)	867 (12.1%)
South Iceland	2212 (7.5%)	1631 (7.3%)	581 (8.1%)
Southern Peninsula	1493 (5.0%)	1019 (4.5%)	474 (6.6%)
Westfjords	457 (1.5%)	324 (1.4%)	133 (1.9%)
West Iceland	1456 (4.9%)	1068 (4.8%)	388 (5.4%)
Living abroad	436 (1.5%)	335 (1.5%)	101 (1.4%)
Unknown	*48*	*33*	*15*
Response period			
Summer (June–Sep)	6026 (20.3%)	4753 (21.1%)	1273 (17.7%)
Fall (Oct–Nov)	4207 (14.2%)	3176 (14.1%)	1031 (14.3%)
Winter (Dec–Mar)	15 419 (51.9%)	11 479 (51.1%)	3940 (54.7%)
Spring (Apr–May)	4029 (13.6%)	3076 (13.7%)	953 (13.2%)
Body max index (BMI)			
Mean (SD)	27.9 (6.0)	27.5 (5.8)	29.1 (6.6)
Normal weight (18.5–24.9)	9532 (35.8%)	7698 (38.1%)	1834 (28.6%)
Underweight (<18.5)	275 (1.0%)	204 (1.0%)	71 (1.1%)
Overweight (25.0–29.9)	8635 (32.4%)	6644 (32.9%)	1991 (31.0%)
Obesity (>30)	8194 (30.8%)	5669 (28.0%)	2525 (39.3%)
Unknown	*3045*	*2269*	*776*
Smoking			
Never	12 709 (47.7%)	10 411 (51.5%)	2298 (35.8%)
Previous smoker	9795 (36.8%)	7150 (35.4%)	2645 (41.2%)
Non-daily smoker	1579 (5.9%)	1134 (5.6%)	445 (6.9%)
Daily smoker	2547 (9.6%)	1516 (7.5%)	1031 (16.1%)
Unknown	*3051*	*2273*	*778*
Binge drinking in past year^‡^			
Never	12 620 (47.4%)	9643 (47.7%)	2977 (46.5%)
Less than once a month	10 301 (38.7%)	7952 (39.3%)	2349 (36.7%)
Monthly	2704 (10.2%)	1993 (9.9%)	711 (11.1%)
Once or more a week	996 (3.7%)	636 (3.1%)	360 (5.6%)
Unknown	*3060*	*2260*	*800*
Leisure based screen time per day			
<3 h	13 890 (47.0%)	11 105 (49.6%)	2785 (39.1%)
3–5 h	8717 (29.5%)	6451 (28.8%)	2266 (31.8%)
5–7 h	3503 (11.9%)	2475 (11.1%)	1028 (14.4%)
>7 h	3418 (11.6%)	2367 (10.6%)	1051 (14.7%)
Unknown	*153*	*86*	*67*
Depressive symptoms^§^			
None/mild	18 164 (70.9%)	15 985 (81.1%)	2179 (36.8%)
Moderate	3952 (15.4%)	2429 (12.3%)	1523 (25.7%)
Moderately severe/severe	3503 (13.7%)	1287 (6.5%)	2216 (37.4%)
Unknown	*4062*	*2783*	*1279*
Anxiety symptoms^‖^			
None/mild	20 426 (77.4%)	17 049 (84.7%)	3377 (53.8%)
Moderate	3547 (13.4%)	2056 (10.2%)	1491 (23.7%)
Severe	2429 (9.2%)	1016 (5.0%)	1413 (22.5%)
Unknown	*3279*	*2363*	*916*

*Expressed as mean (*SD*) for continuous variables and proportions for categorical variables. n: sample size.

^†^Active: working, studying or on parental leave; inactive: on disability, sick leave, unemployed, or retired.

^‡^Six or more drinks on one occasion (one drink is defined as simple measure of spirits, one glass of wine or one small beer).

^§^PHQ-9; symptoms past 2 weeks.

^‖^GAD-7; symptoms past 2 weeks.

### Sleep disturbances

The mean PSQI global score was 7.7 (±4.0). The majority (65.5%) of the women experienced sleep problems in the past month (PSQI > 5), and nearly a quarter (24.2%) had severe sleep problems (PSQI > 10) (Table 2). Nearly half of the women (47.5%) slept less than seven hours each night in the past month and 44.9% reported sleep latency greater than 30 min at least once a week in the past month. Furthermore, 40.5% of women reported moderate to severe sleep disturbances, for example, waking up in the middle of the night or early morning, coughing or snoring loudly, or feeling too cold or hot. Approximately one-third (32.8%) of women spent ≤74% of time asleep while in bed and 32.4% rated their overall sleep quality during the past month as fair or very poor. One-fifth (20.3%) of women had moderate to severe daytime dysfunction and one-third (32.3%) reported using over the counter or prescribed sleep medication in the past month (Table 2; Supplementary Tables S1 and S2).

**Table 2.** Overall score and components on the PSQI

	Overall (*n* = 29 681)*
PSQI overall score	
Sleep problems (PSQI > 5)	19 437 (65.5%)
Severe sleep problems (PSQI > 10)	7197 (24.2%)
PSQI components	
1. Fairly or very poor overall sleep quality	9608 (32.4%)
2. Sleep latency > 30 min	13 338 (44.9%)
3. Sleep duration <7 h	14 110 (47.5%)
4. Habitual sleep efficiency ≤ 74%	9734 (32.8%)
5. Moderate to severe sleep disturbances	12 015 (40.5%)
6. Use of sleep medication^†^	9588 (32.3%)
7. Moderate to severe daytime dysfunction	6020 (20.3%)

*Expressed as proportions.

^†^Prescription or over the counter.

### Social determinants of severe sleep problems

**Figure 1.**
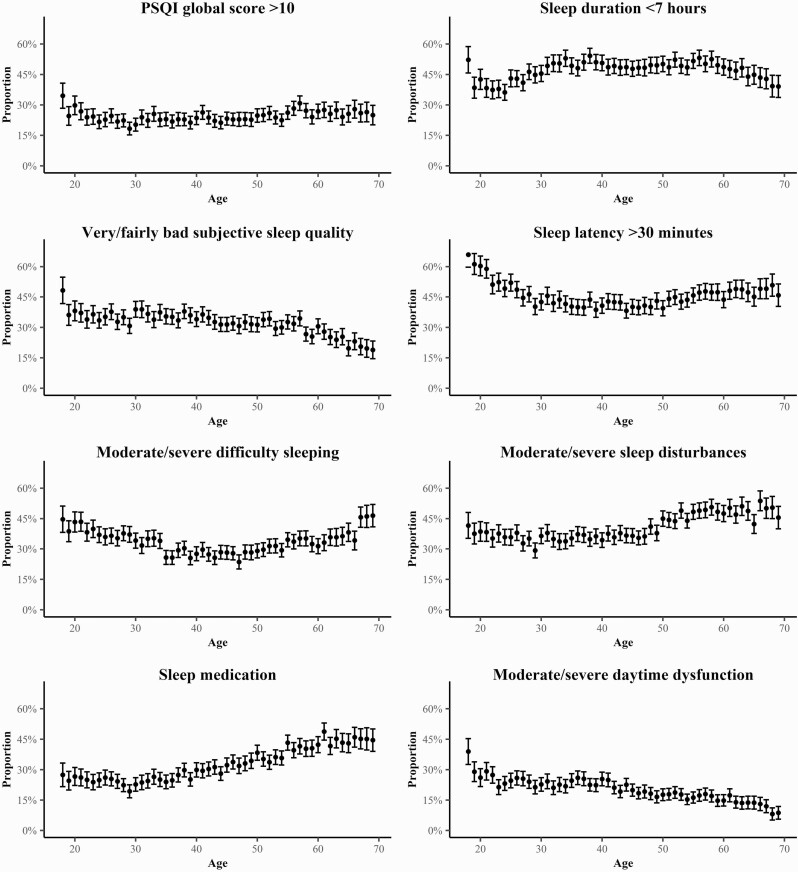
Association between age, severe sleep problems (PSQI > 10), and the PSQI components. The dots represent the proportion of women in each age category experiencing severe sleep problems and lines represent 95% confidence intervals.

We found a U-shaped association between age and severe sleep problems (PSQI > 10). When assessing the association between individual PSQI components and age we also found a U-shaped relationship between age and both sleep latency >30 min and moderate/severe difficulty sleeping. In contrast, we found an inverted U-shaped association between age and sleep duration <7 h. Further, the association between age and both sleep medication use and moderate/severe sleep disturbances was positive, while a negative association was found between age and moderate/severe daytime dysfunction and fairly/very bad subjective sleep quality (Figure 1).

**Figure 2.**
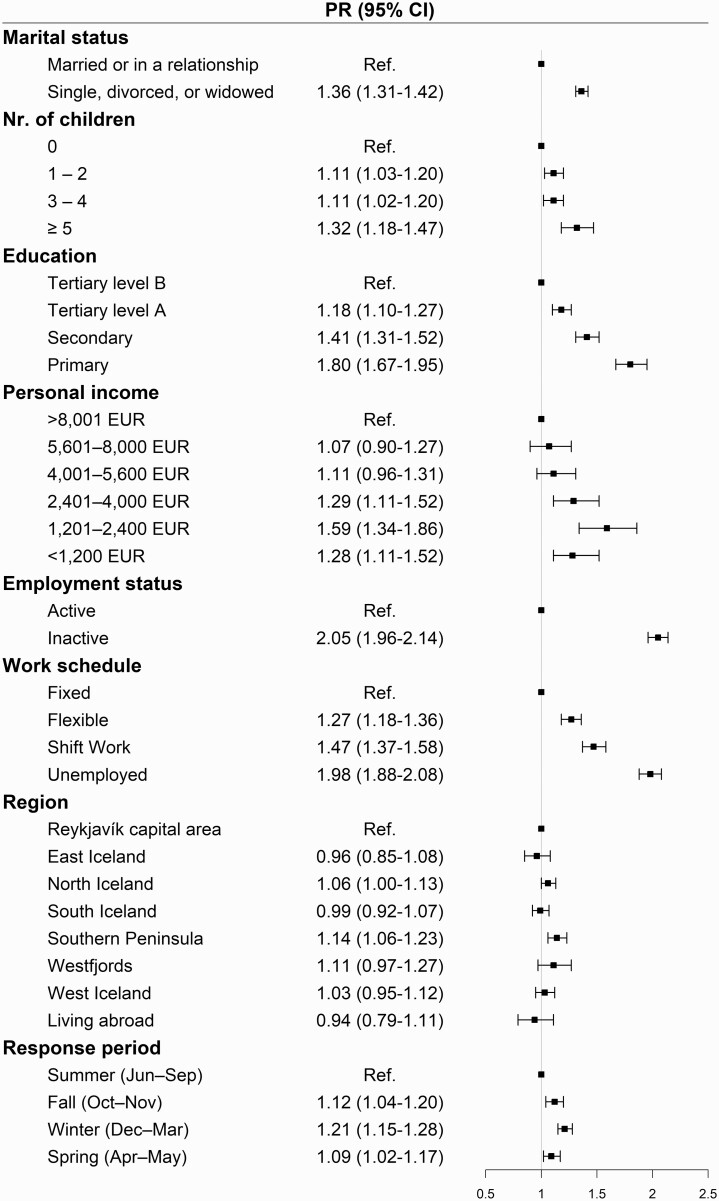
Association of demographic characteristics and severe sleep problems during the past month (PSQI > 10)*. *Expressed as proportions and as prevalence ratio (PR) with 95% confidence intervals (CIs). PRs are adjusted for age, marital status, number of children, education, personal income, work schedule, region, and response period.

We found being single, divorced, or widowed to be associated with severe sleep problems, compared to women married or in a relationship (adjusted PR [aPR], 1.36; 95% CI = 1.31 to 1.42). Furthermore, compared to having no children, women with one or two (aPR, 1.11; 95% CI, 1.03 to 1.20), three or four (aPR, 1.11; 95% CI, 1.02–1.20), or five or more children (aPR, 1.32; 95% CI, 1.18 to 1.47) had higher prevalence of severe sleep problems. Less education was associated with higher prevalence of severe sleep problems as well as low personal income. Being inactive in the labor market had a strong association with severe sleep problems (aPR, 2.05; 95% CI, 1.96 to 2.14). Having a flexible work schedule (aPR, 1.27; 95% CI, 1.18 to 1.36), working shifts (aPR, 1.47; 95% CI, 1.37 to 1.58), or being unemployed (aPR, 1.98; 95% CI, 1.88 to 2.08) was associated with higher prevalence of severe sleep problems compared to having a fixed work schedule (Figure 2).

### Geographic location, seasons, and severe sleep problems

**Figure 3.**
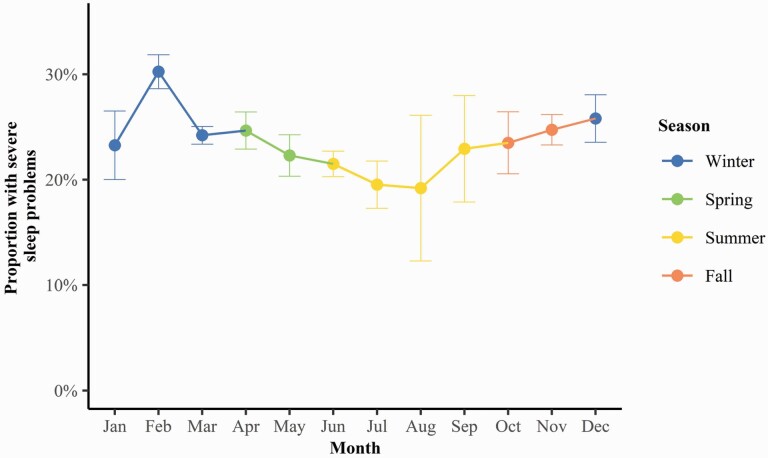
Association between response period and severe sleep problems (PSQI > 10). The dots represent the proportion of women in each month experiencing severe sleep problems in the preceding month and lines represent 95% confidence intervals.

There were some geographic differences in severe sleep problems observed. Compared to living in the Reykjavik capital area, living in Southern Peninsula (aPR, 1.14; 95% CI, 1.06 to 1.23) and North Iceland (aPR, 1.06; 95% CI, 1.00 to 1.13) was associated with higher prevalence of severe sleep problems (Figure 2). Compared to women who participated in the study during the summer, women who responded during the winter (aPR, 1.21; 95% CI, 1.15 to 1.28), fall (aPR, 1.12; 95% CI, 1.04 to 1.20), and spring (aPR 1.09; 95% CI, 1.02 to 1.17) had a higher prevalence of severe sleep problems (Figures 2 and 3). When analyzing the association of PSQI components and response period, we found the prevalence to be highest during winter on all components, except for sleep duration and sleep latency (Supplementary Table S3).

### Health-related behavior, well-being, and severe sleep problems

**Figure 4.**
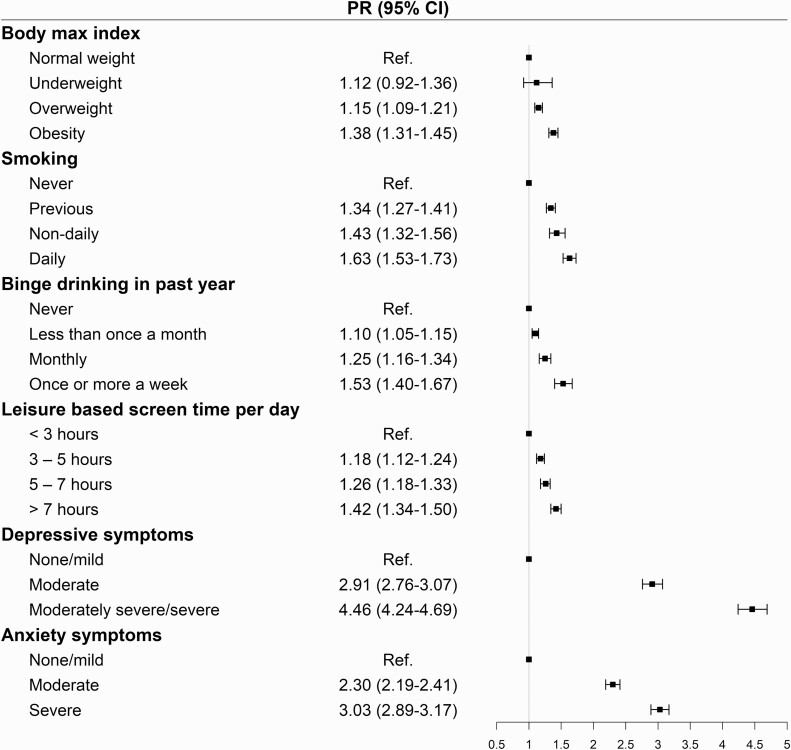
Associations of BMI, health-related behavior, and well-being with severe sleep problems during the past month (PSQI > 10)*. *Expressed as proportions and as prevalence ratio (PR) with 95% confidence intervals (CIs). PRs are adjusted for age, marital status, number of children, education, personal income, work schedule, region, and response period.

Suboptimal health-related behavior (i.e. smoking and binge drinking) and BMI were associated with higher prevalence of severe sleep problems (Figure 4). Particularly, being overweight (aPR, 1.15; 95% CI, 1.09 to 1.21) or obese (aPR, 1.38; 95% CI, 1.31 to 1.45) was associated with higher prevalence of severe sleep problems compared to being normal weight. Daily smokers (aPR, 1.63; 95% CI, 1.53 to 1.73), non-daily smokers (aPR, 1.43; 95% CI, 1.32 to 1.56), and previous smokers (aPR, 1.34; 95% CI, 1.27 to 1.41) had higher prevalence of severe sleep problems compared to women who have never smoked. Moreover, we found that severe sleep problems increased as a function of increased binge drinking. The association between daily leisure-based screen time and severe sleep problems was statistically significant, that is, increased screen time was associated with higher prevalence of severe sleep problems (Figure 4).

Compared to women with no or mild depressive symptoms, women with moderate (aRR, 2.91; 95% CI, 2.76 to 3.07) and moderately severe/severe (aRR, 4.46; 95% CI, 4.24 to 4.69) depressive symptoms had a higher prevalence of severe sleep problems. Similarly, women with moderate (aPR, 2.30; 95% CI, 2.19 to 2.41) and severe (aPR, 3.03; 95% CI, 2.89 to 3.17) anxiety symptoms had a higher prevalence of severe sleep problems, compared to women with none or mild anxiety symptoms (Figure 4).

### Additional results

Education, personal income, employment status, work schedule, BMI, smoking, and depressive- and anxiety symptoms were effect modifiers when looking at the association between severe sleep problems and age groups (i.e., young age [18–39], middle-aged [40–59], and old [60+]). We found that low education, being inactive in the labor market, smoking, and well-being (i.e. symptoms of depression and anxiety) were associated with higher prevalence of severe sleep problems among women of young age compared to those who were middle aged or old. Additionally, low income and working shifts were associated with higher prevalence of severe sleep problems of middle aged and old women compared to those who were young (Supplementary Table S4).

The association between the independent variables and severe sleep problems during the past month (PSQI > 10) were similar using pooled data after multiple imputation (Supplementary Table S5) and complete cases (Supplementary Table S6). In the current study, the internal consistency of the seven PSQI components was acceptable (Cronbach’s α = .78).

## Discussion

This population-based study among women in Subarctic regions indicates that one in five experience severe sleep problems, particularly during wintertime. We further found U-shaped relationship between age and severe sleep problems, with severe sleep problems being more common among women in their 20s and late 50s compared to middle-aged women. Being single, divorced, or widowed, having children, low SES, unemployment, working flexible hours, and shift work were associated with higher prevalence of severe sleep problems. Health-related factors were also associated with severe sleep problems including being overweight or obese, smoking, binge drinking, and excessive leisure-based screen time.

In our study, approximately half of women are not getting the recommended amount of sleep (i.e. at least 7 h) and many experiences prolonged sleep latency. These results are in line with a previous study that found Icelandic adolescents to have delayed bedtimes and shorter sleep duration compared to their European peers [55]. Our results indicate that sleeping problems are more prevalent among Icelandic women than women in other countries with 65% of women experience sleep problems (PSQI > 5), compared to 27%–53% of women in the United States [7], Germany [8], Korea [9], Spain [10], China [11], and Hong Kong [12]. This is possibly due to greater seasonal changes in light exposure and increased exposure to artificial light at night during the short photoperiod of the Arctic winters [20–22, 56]. Indeed, we found that women who reported their sleep quality during the fall and winter had 12%–21% higher prevalence of severe sleep problems compared to women who participated in the summer and also the highest prevalence of severe sleep problems on all PSQI components, except sleep duration. This is consistent with results of a previous study finding that insomnia and fatigue were more common in January than in August among residents of Northern Norway (69°N), while only small seasonal differences in sleep were found among individuals living in Ghana (5°N) [21]. Previous studies have also found seasonal variation in light exposure to be associated with disturbed circadian system [20], rise- and bedtime, and sleep efficiency [21, 22]. In addition, insomnia appears to be more common among women in Subarctic regions [16–18] than women residing elsewhere [19].

We found severe sleep problems to be more prevalent among young and late-midlife women compared to middle-aged women. Previous studies exploring the relationship between age and sleep problems have found conflicting results. While some studies found steady worsening of sleep problems with increasing age, others found sleep problems to increase in late midlife [10, 57]. For instance, it has been indicated that menopause negatively impacts sleep, independent of other factors such as age [58]. Other study has found younger women to report high prevalence of self-reported sleep disturbances [59]. A possible explanation of the discrepancy in prior studies is that the association between sleep and age differs for different aspects of sleep. Indeed, we found that sleep medication uses and sleep disturbances were more common among late-midlife women while prolonged sleep latency, poor subjective sleep quality, and daytime dysfunction were more common among young women.

We found that 36% of women aged 18–29 years reported spending five hours or more in front of screens daily, compared to 20% of women of other ages (*p* < .001). Many factors can negatively impact young adults’ sleep, especially the increased social media usage in recent years [60]. Research has shown that smartphones can disrupt sleep through artificial short-waved blue light exposure, which may affect a malfunction of the circadian timing system and melatonin levels [61]. In addition, unhealthy lifestyle, such as inadequate physical activity, alcohol-, and nicotine use can negatively affect sleep [62].

In line with previous research [24, 25], Subarctic women living alone had a higher prevalence of severe sleep problems compared to those in a relationship. This is possibly due to the positive influence of social support and relationships on sleep [63]. Moreover, we found a higher prevalence of severe sleep problems among women who had children. Previous research indicates that parents report more sleep disturbances than childless adults [26]. Interestingly, studies have also found a positive association between children’s sleep disruptions and poor sleep quality among parents, regardless of children’s age [27].

Overall, we found that socioeconomic hardship and working flexible hours or working shifts were associated with higher prevalence of severe sleep problems among women living in Subarctic regions. These results are consistent with previous studies which have found low SES and unemployment to be associated with higher prevalence of sleep problems [7, 8, 28, 29]. This is possibly due to stress resulting from financial strain, which is associated with both low SES, and sleep problems among women [64]. Indeed, we found that women who were currently inactive in the labor market were more likely to report low income compared to women active in the labor market (62% vs. 25%; *p* < .001). Previous studies have found shift work to be associated with sleep problems [30, 31], especially among women and older adults [65]. Shift work can have a disruptive effect on normal circadian rhythms and result in physiological stress and chronic impairment of cognition [31]. These results indicate the need to target women with low SES and those working shifts when promoting interventions for improved sleep.

Compared to the Reykjavik capital area (64°08.5′N, 21°55.6′V), living in the Southern Peninsula (e.g. Keflavík: 64°00.2′N, 22°33.9′V) was associated with severe sleep problems. Nearly one-third of residents of the Southern Peninsula are experiencing severe sleep problems. This is the region with the greatest socioeconomic- and public health challenges in the country, such as the lowest prevalence of university-educated inhabitants and the highest prevalence of smoking, obesity (BMI >30), and poor physical and mental health [66]; all factors which have been associated with sleep problems. Therefore, further studies assessing the effect of living in Subarctic regions on sleep are needed.

We further found that high BMI and poor health behaviors, such as cigarette smoking, excessive alcohol consumption, and prolonged leisure-based screen time, were associated with higher prevalence of severe sleep problems among women living in Subarctic regions. Previous studies have found obesity to be associated with a higher prevalence of severe sleep problems, compared to being normal weight [8, 32, 33]. This relationship is bidirectional as sleep deprivation can inhibit the production of the hormone leptin which regulates food intake [67] and obesity is a risk factor for the development of obstructive sleep apnea [68]. The relationship between smoking and sleep problems is also well studied on a population level [8, 9, 34, 35]. Further, excessive alcohol consumption has been associated with sleep problems [36, 37], such as sleep continuity and prolonged sleep latency, during the first half of the night, and increased wakefulness, rapid eye movement rebound, and early morning awakenings during the second half of the night [69–71]. Further, our finding that prolonged screen time was associated with severe sleep problems is in line with previous studies suggesting that increased screen time is associated with longer sleep latency, reduced sleep duration, and decreased sleep quality [38–40]. Besides the potential impact of BMI, smoking, binge-drinking, and leisure-based screen time on sleep quality, prior research has also suggested that the onset of disturbed sleep, in turn, can lead to adverse changes in health-related behavior, such as increased risk of alcohol use, smoking, physical inactivity, and overweight or obesity [72].

Consistent with previous research [41], we found a strong association between severe sleep problems and anxiety and depressive symptoms. While insomnia and hypersomnia are symptoms of depression, insomnia has also been found to increase the risk of depressive symptoms, such as suicidal ideation [73, 74]. Therefore, treating sleep problems can be an important step in reducing the risk of subsequent mental health difficulties.

This study has several strengths worth mentioning. First, the SAGA cohort is a large study with over 30 000 participants who are representative of Icelandic women with regard to age, education, income, and residency [44]. It is also the first nationwide, population-based study to investigate a wide range of sleep problems and associated factors among women residing in Subarctic region. Secondly, the measurement of sleep problems used in the study has been validated in different countries, languages, and samples and is a reliable measurement tool.

Several limitations need to be recognized. First, considering that only women between the ages of 18 and 69 were selected for the study, it is not possible to generalize our results to males or to women in other age groups. Second, this study was cross-sectional, making it impossible to infer the causality of the studied associations. Third, there might be significant differences between objective and self-reported measures of sleep, and this study only included the latter.

In conclusion, our results indicate that the prevalence of sleep problems in the general population of Icelandic women is higher than in other international population-based samples of women. We found higher prevalence of severe sleep problems among women responding during the winter months, when sunlight is limited. In addition, we also confirmed that socioeconomic challenges, shift work, suboptimal health behaviors, and mental health problems are also associated with elevated risks of sleep problems among women in Subarctic region of Iceland with high-social welfare. These results are valuable for identifying women at greatest risk of sleep disruptions and would therefore benefit from targeted prevention, in the Subarctic.

## Supplementary Material

zsac100_suppl_Supplementary_Material

## Data Availability

The data underlying this article are not currently publicly available due to data protection laws but may be available from the corresponding author on reasonable request.
