# Supplementary material for: Sleep disturbances among women in a Subarctic region: a nationwide study
Source: Sleep. 2022 May 9;45(8):zsac100. doi: 10.1093/sleep/zsac100 (PMC9366651; doi:10.1093/sleep/zsac100)
Supplement: zsac100_suppl_Supplementary_Material [file zsac100_suppl_supplementary_material.docx]

Sleep Disturbances among Women in a Subarctic region:

A Nationwide Study

Anna Bára Unnarsdóttir^1^, Arna Hauksdóttir^1^, Thor Aspelund^1^, Vigdís Gunnarsdóttir^1^, Gunnar Tómasson^1,2^, Jóhanna Jakobsdóttir^1^, Unnur Anna Valdimarsdóttir^1,3,4^, Edda Bjork Thordardottir^1^.

^1 Centre of Public Health Sciences, Faculty of Medicine, School of Health Sciences, University of Iceland, Reykjavik, Iceland.
2 Department of Rheumatology, University Hospital, Iceland.
3 Department of Medical Epidemiology and Biostatistics, Karolinska Institutet, Stockholm, Sweden.^  ^4 Department of Epidemiology, Harvard TH Chan School of Public Health, Boston, Massachusetts, USA.^

**Corresponding author**

Anna Bára Unnarsdóttir, [abu@hi.is](mailto:abu@hi.is)

Centre of Public Health Sciences, Faculty of Medicine, University of Iceland

Sturlugata 8, 101 Reykjavik, Iceland

**Supplementary Materials**

**Table S1.** Counts and cumulative percentages of the PSQI
total scores

| **PSQI total score** | **Count** | **Cumulative count** | **Cumulative percentage** |
| --- | --- | --- | --- |
| **0** | 134 | 134 | 0.4% |
| **1** | 620 | 754 | 2.5% |
| **2** | 1,327 | 2,081 | 7.0% |
| **3** | 2,202 | 4,283 | 14.4% |
| **4** | 2,895 | 7,178 | 24.2% |
| **5** | 3,066 | 10,244 | 34.5% |
| **6** | 2,959 | 13,203 | 44.5% |
| **7** | 2,782 | 15,985 | 53.9% |
| **8** | 2,472 | 18,457 | 62.2% |
| **9** | 2,189 | 20,646 | 69.6% |
| **10** | 1,838 | 22,484 | 75.8% |
| **11** | 1,630 | 24,114 | 81.2% |
| **12** | 1,419 | 25,533 | 86.0% |
| **13** | 1,203 | 26,736 | 90.1% |
| **14** | 961 | 27,697 | 93.3% |
| **15** | 690 | 28,387 | 95.6% |
| **16** | 520 | 28,907 | 97.4% |
| **17** | 364 | 29,271 | 98.6% |
| **18** | 198 | 29,469 | 99.3% |
| **19** | 139 | 29,608 | 99.8% |
| **20** | 60 | 29,668 | 100.0% |
| **21** | 13 | 29,681 | 100.0% |

**Table S2.** Frequencies of responses on the seven PSQI components scores.

| **PSQI components** | **PSQI items** | **Response options** | ***n*** | **%** |
| --- | --- | --- | --- | --- |
| **Subjective sleep quality** | #6 During the past month, how would you rate your sleep quality overall? | Very good (0) | 3,947 | 13.3% |
|  |  | Fairly good (1) | 16,126 | 54.3% |
|  |  | Fairly bad (2) | 7,916 | 26.7% |
|  |  | Very bad (3) | 1,692 | 5.7% |
| **Sleep latency^a^** | #2 During the past month, how long (in minutes) has it usually take you to call asleep each night?  #5a During the past month, how often have you had trouble sleeping because you cannot get to sleep within 30 minutes | $\leq$ 15 minutes / Not during the past month (0) | 5,913 | 19.9% |
|  |  | 16$-$30 minutes / Less than once a week (1) | 10,430 | 35.1% |
|  |  | 31$-$60 minutes / Once or twice a week (2) | 6,907 | 23.3% |
|  |  | > 60 minutes / Three or more times a week (3) | 6,431 | 21.7% |
| **Sleep duration** | #4 During the past month, how many hours of actual sleep did you get at night? | > 7 hours (0) | 15,571 | 52.5% |
|  |  | 6$-$7 hours (1) | 9,639 | 32.5% |
|  |  | 5$-$6 hours (2) | 3,355 | 11.3% |
|  |  | < 5 hours (3) | 1,116 | 3.8% |
| **Habitual sleep efficiency** | #4 Number of hours slept / #3-#1 Number of hours in bed x 100 | > 85% (0) | 8,391 | 28.3% |
|  |  | 75$-$84% (1) | 11,556 | 38.9% |
|  |  | 65$-$74% (2) | 5,426 | 18.3% |
|  |  | < 65% (3) | 4,308 | 14.5% |
| **Sleep disturbances** | #5b-5j During the past month, how often have you had trouble sleeping because you b) wake up in the middle of the night or early morning, c) have to get up to use the bathroom, d) cannot breathe comfortably, e) cough or snore loudly, f) feel too cold, g) feel too hot, h) had bad dreams, i) have pain, j) other reasons. | Not during the past month (0)  Less than once a week (1)  Once or twice a week (2)  Three or more times a week (3) | 1,186 | 4.0% |
|  |  |  | 16,480 | 55.5% |
|  |  |  | 10,439 | 35.2% |
|  |  |  | 1,576 | 5.3% |
| **Use of sleep medication** | #7 During the past month, how often have you taken medication (prescribed or “over the counter”) to help you sleep? | Not during the past month (0) | 20,093 | 67.7% |
|  |  | Less than once a week (1) | 2,954 | 10.0% |
|  |  | Once or twice a week (2) | 1,634 | 5.5% |
|  |  | Three or more times a week (3) | 5,000 | 16.8% |
| **Daytime dysfunction^b^** | #8 During the past month, how often have you had trouble staying awake while driving, eating meals, or engaging in social activity?  #9 During the past month, how much of a problem has it been for you to keep up enough enthusiasm to get things done? | Never / No problem at all (0) | 7,173 | 24.2% |
|  |  | Once or twice / Only a very slight problem (1) | 16,488 | 55.6% |
|  |  | Once or twice each week /  Somewhat of a problem (2) | 4,994 | 16.8% |
|  |  | Three or more times each week /  A very big problem (3) | 1,026 | 3.5% |

^a^ Sum of items #2 and #5a on the PSQI were used to find the components score, i.e., 0=0, 1-2=1, 3-4=2, 5-6=3.
^b^ Sum of items #8 and 9 on the PSQI were used to find the components score, i.e., 0=0, 1-2=1, 3-4=2, 5-6=3.

| **Table S3.** Association of PSQI components and season. | | | | | |
| --- | --- | --- | --- | --- | --- |
| **PSQI components** | **Season** | | | | ***p*-value** |
|  | **Summer** (n=6,026) | **Fall** (n=4,207) | **Winter** (n=15,419) | **Spring** (n=4,029) |  |
| 1. Fairly or very poor overall sleep quality | 1,749 (29.0%) | 1,401 (33.3%) | 5,238 (34.0%) | 1,220 (30.3%) | <.001 |
| 2. Sleep latency >30 minutes | 2,566 (42.6%) | 1,953 (46.4%) | 7,098 (46.0%) | 1,721 (42.7%) | <.001 |
| 3. Sleep duration <7 hours | 2,832 (47.0%) | 1,994 (47.4%) | 7,385 (47.9%) | 1,899 (47.1%) | .616 |
| 4. Habitual sleep efficiency $\leq$74% | 1,804 (29.9%) | 1,407 (33.4%) | 5,239 (34.0%) | 1,284 (31.9%) | <.001 |
| 5. Moderate to severe sleep disturbances | 2,337 (38.8%) | 1,696 (40.3%) | 6,368 (41.3%) | 1,614 (40.1%) | .008 |
| 6. Use of sleep medication | 1,856 (30.8%) | 1,273 (30.3%) | 5,135 (33.3%) | 1,324 (32.9%) | <.001 |
| 7. Moderate to severe daytime dysfunction | 1,081 (17.9%) | 827 (19.7%) | 3,294 (21.4%) | 818 (20.3%) | <.001 |

| **Table S4.** The association between severe sleep problems and demographic-and socioeconomic characteristics, health-behaviors, and wellbeing, stratified by age groups. | | | |
| --- | --- | --- | --- |
|  | **Young age (18-39) (n=9,087)** | **Middle age (40-59) (n=10,611)** | **Old age (60+) (n=3,445)** |
|  | **PR (95% CI)^a^** | **PR (95% CI)^a^** | **PR (95% CI)^a^** |
| **Education** |  |  |  |
| Tertiary level B | 1 (ref.) | 1 (ref.) | 1 (ref.) |
| Tertiary level A | **1.20 (1.04-1.39)** | **1.19 (1.07-1.33)** | 1.11 (0.89-1.37) |
| Secondary | **1.51 (1.30-1.75)** | **1.26 (1.13-1.42)** | 1.15 (0.93-1.43) |
| Primary | **2.29 (1.97-2.67)** | **1.44 (1.26-1.63)** | **1.26 (1.00-1.59)** |
| **Personal income** |  |  |  |
| >8,001 EUR | 1 (ref.) | 1 (ref.) | 1 (ref.) |
| 5,601–8,000 EUR | 1.02 (0.70-1.48) | 1.14 (0.90-1.45) | 1.75 (0.86-3.55) |
| 4,001–5,600 EUR | 0.94 (0.66-1.32) | 1.21 (0.97-1.51) | 1.66 (0.83-3.29) |
| 2,401–4,000 EUR | 1.21 (0.87-1.69) | **1.49 (1.19-1.87)** | 1.66 (0.84-3.28) |
| 1,201–2,400 EUR | 1.38 (0.99-1.93) | **1.75 (1.38-2.20)** | **2.33 (1.18-4.62)** |
| <1,200 EUR | 0.98 (0.69-1.38) | **1.33 (1.00-1.79)** | **2.63 (1.27-5.45)** |
| **Employment status** |  |  |  |
| Active | 1 (ref.) | 1 (ref.) | 1 (ref.) |
| Inactive | **1.85 (1.66-2.07)** | **1.58 (1.31-1.91)** | 1.35 (0.83-2.20) |
| **Work Schedule** |  |  |  |
| Fixed | 1 (ref.) | 1 (ref.) | 1 (ref.) |
| Flexible | **1.40 (1.21-1.61)** | **1.22 (1.08-1.38)** | 1.12 (0.87-1.46) |
| Shift Work | **1.35 (1.19-1.53)** | **1.61 (1.43-1.82)** | **1.51 (1.19-1.91)** |
| Unemployed | **1.81 (1.64-1.99)** | **2.22 (2.03-2.43)** | **1.53 (1.31-1.78)** |
| **Body max index** |  |  |  |
| Normal weight | 1 (ref.) | 1 (ref.) | 1 (ref.) |
| Underweight | 1.06 (0.79-1.42) | 1.02 (0.69-1.53) | 0.86 (0.41-1.80) |
| Overweight | 1.08 (0.98-1.19) | **1.21 (1.10-1.32)** | 1.09 (0.94-1.27) |
| Obesity | **1.47 (1.34-1.60)** | **1.41 (1.29-1.54)** | **1.20 (1.03-1.40)** |
| **Smoking** |  |  |  |
| Never | 1 (ref.) | 1 (ref.) | 1 (ref.) |
| Previous | **1.60 (1.46-1.75)** | **1.21 (1.12-1.31)** | **1.22 (1.06-1.41)** |
| Non-daily | **1.59 (1.40-1.82)** | **1.24 (1.06-1.44)** | **1.45 (1.08-1.93)** |
| Daily | **1.92 (1.72-2.14)** | **1.48 (1.34-1.64)** | **1.48 (1.21-1.80)** |
| **Depressive symptoms** |  |  |  |
| None/mild | 1 (ref.) | 1 (ref.) | 1 (ref.) |
| Moderate | **3.36 (3.00-3.76)** | **2.99 (2.74-3.26)** | **2.62 (2.29-2.99)** |
| Moderately severe/   severe | **5.80 (5.24-6.43)** | **4.20 (3.87-4.55)** | **3.12 (2.72-3.57)** |
| **Anxiety symptoms** |  |  |  |
| None/mild | 1 (ref.) | 1 (ref.) | 1 (ref.) |
| Moderate | **2.55 (2.32-2.80)** | **2.22 (2.06-2.41)** | **2.13 (1.84-2.47)** |
| Severe | **3.56 (3.25-3.88)** | **2.67 (2.46-2.89)** | **2.71 (2.31-3.18)** |
| ^a^ The prevalence ratios (PRs) were adjusted for marital status, nr. of children, education, personal income, work schedule, region, and response period. | | | |

**Table S5.** Association of severe sleep problems during the past month (PSQI >10)
and demographic characteristics, BMI, health-related behaviour, and daily
leisure-based screen time, using pooled data after multiple imputation.*

|  | PR (95% CI)^a^ | PR (95% CI)^b^ |
| --- | --- | --- |
| **Marital status** |  |  |
| Married or in a relationship | 1 (ref.) | 1 (ref.) |
| Single, divorced, or widowed | **1.46 (1.40-1.52)** | **1.36 (1.31-1.42)** |
| **Nr. of children** |  |  |
| 0 | 1 (ref.) | 1 (ref.) |
| 1 – 2 | 1.00 (0.93-1.07) | **1.11 (1.03-1.20)** |
| 3 – 4 | 1.02 (0.94-1.10) | **1.11 (1.02-1.20)** |
| ≥ 5 | **1.36 (1.22-1.52)** | **1.32 (1.18-1.47)** |
| **Education** |  |  |
| Tertiary level B | 1 (ref.) | 1 (ref.) |
| Tertiary level A | **1.35 (1.26-1.45)** | **1.18 (1.10-1.27)** |
| Secondary | **1.88 (1.75-2.01)** | **1.41 (1.31-1.52)** |
| Primary | **2.62 (2.45-2.81)** | **1.80 (1.67-1.95)** |
| **Personal income** |  |  |
| >8,001 EUR | 1 (ref.) | 1 (ref.) |
| 5,601–8,000 EUR | 1.09 (0.92-1.29) | 1.07 (0.90-1.27) |
| 4,001–5,600 EUR | **1.21 (1.03-1.42)** | 1.11 (0.96-1.31) |
| 2,401–4,000 EUR | **1.74 (1.48-2.03)** | **1.29 (1.11-1.52)** |
| 1,201–2,400 EUR | **2.86 (2.45-3.34)** | **1.59 (1.34-1.86)** |
| <1,200 EUR | **2.39 (2.02-2.83)** | **1.28 (1.11-1.52)** |
| **Employment status** |  |  |
| Active | 1 (ref.) | 1 (ref.) |
| Inactive | **2.62 (2.51-2.73)** | **2.05 (1.96-2.14)** |
| **Work schedule** |  |  |
| Fixed | 1 (ref.) | 1 (ref.) |
| Flexible | **1.30 (1.21-1.40)** | **1.27 (1.18-1.36)** |
| Shift Work | **1.73 (1.61-1.85)** | **1.47 (1.37-1.58)** |
| Unemployed | **2.44 (2.33-2.55)** | **1.98 (1.88-2.08)** |
| **Region** |  |  |
| Reykjavík capital area | 1 (ref.) | 1 (ref.) |
| East Iceland | 1.10 (0.96-1.24) | 0.96 (0.85-1.08) |
| North Iceland | **1.24 (1.17-1.32)** | **1.06 (1.00-1.13)** |
| South Iceland | **1.16 (1.08-1.25)** | 0.99 (0.92-1.07) |
| Southern Peninsula | **1.41 (1.30-1.53)** | **1.14 (1.06-1.23)** |
| Westfjords | **1.28 (1.11-1.48)** | 1.11 (0.97-1.27) |
| West Iceland | **1.18 (1.08-1.29)** | 1.03 (0.95-1.12) |
| Living abroad | 1.05 (0.88-1.25) | 0.94 (0.79-1.11) |
| **Response period** |  |  |
| Summer (Jun–Sep) | 1 (ref.) | 1 (ref.) |
| Fall (Oct–Nov) | **1.17 (1.09-1.26)** | **1.12 (1.04-1.20)** |
| Winter (Dec–Mar) | **1.22 (1.16-1.29)** | **1.21 (1.15-1.28)** |
| Spring (Apr–May) | **1.12 (1.04-1.20)** | **1.09 (1.02-1.17)** |
| **Body max index** |  |  |
| Normal weight | 1 (ref.) | 1 (ref.) |
| Underweight | **1.30 (1.06-1.59)** | 1.12 (0.92-1.36) |
| Overweight | **1.19 (1.13-1.26)** | **1.15 (1.09-1.21)** |
| Obesity | **1.60 (1.52-1.68)** | **1.38 (1.31-1.45)** |
| **Smoking** |  |  |
| Never | 1 (ref.) | 1 (ref.) |
| Previous | **1.49 (1.42-1.57)** | **1.34 (1.27-1.41)** |
| Non-daily | **1.57 (1.44-1.72)** | **1.43 (1.32-1.56)** |
| Daily | **2.23 (2.10-2.36)** | **1.63 (1.53-1.73)** |
| **Binge drinking in past year** |  |  |
| Never | 1 (ref.) | 1 (ref.) |
| Less than once a month | 1.01 (0.96-1.06) | **1.10 (1.05-1.15)** |
| Monthly | **1.16 (1.08-1.25)** | **1.25 (1.16-1.34)** |
| Once or more a week | **1.52 (1.39-1.66)** | **1.53 (1.40-1.67)** |
| **Leisure based screen time per day** |  |  |
| < 3 hours | 1 (ref.) | 1 (ref.) |
| 3$-$5 hours | **1.32 (1.25-1.38)** | **1.18 (1.12-1.24)** |
| 5$-$7 hours | **1.50 (1.41-1.60)** | **1.26 (1.18-1.33)** |
| > 7 hours | **1.58 (1.49-1.68)** | **1.42 (1.34-1.50)** |
| **Depressive symptoms** |  |  |
| None/mild | 1 (ref.) | 1 (ref.) |
| Moderate | **3.28 (3.11-3.46)** | **2.91 (2.76-3.07)** |
| Moderately severe/   severe | **5.49 (5.24-5.75)** | **4.46 (4.24-4.69)** |
| **Anxiety symptoms** |  |  |
| None/mild | 1 (ref.) | 1 (ref.) |
| Moderate | **2.68 (2.56-2.82)** | **2.30 (2.19-2.41)** |
| Severe | **3.81 (3.64-3.98)** | **3.03 (2.89-3.17)** |

^*^ Expressed as proportions and as prevalence ratio (PR) with 95% confidence
intervals (CI). ^a^ Adjusted for age.
^b^ Adjusted for age, marital status, nr. of children, education, personal income, work schedule, region, and response period.

**Table S6.** Association of severe sleep problems during the past month (PSQI >10) and demographic characteristics, BMI, health-related behaviour, and daily leisure-based screen
time, using complete cases.^*^

|  | PSQI$\leq$10 (n=17,881) | PSQI >10 (n=5,262) | cPR (95% CI)^a^ | aPR (95% CI)^b^ |
| --- | --- | --- | --- | --- |
| **Marital status** |  |  |  |  |
| Married or in a relationship | 14,056 (79.6%) | 3,605 (20.4%) | 1 (ref.) | 1 (ref.) |
| Single, divorced, or widowed | 3,825 (69.8%) | 1,657 (30.2%) | **1.48 (1.41-1.56)** | **1.38 (1.32-1.46)** |
| **Nr. of children** |  |  |  |  |
| 0 | 3,486 (77.4%) | 1,011 (22.5%) | 1 (ref.) | 1 (ref.) |
| 1 – 2 | 7,164(78.0%) | 2,026 (22.0%) | 0.98 (0.90-1.06) | **1.11 (1.11-1.31)** |
| 3 – 4 | 6,588 (77.3%) | 1,937 (22.7%) | 1.00 (0.91-1.09) | **1.11 (1.29-1.52)** |
| ≥ 5  | 643 (69.1%) | 288 (30.9%) | **1.35 (1.19-1.53)** | **1.31 (1.66-1.98)** |
| **Education** |  |  |  |  |
| Tertiary level B | 4,710 (85.5%) | 799 (14.5%) | 1 (ref.) | 1 (ref.) |
| Tertiary level A | 6,067 (80.4%) | 1,483 (19.6%) | **1.38 (1.27-1.49)** | **1.21 (1.11-1.31)** |
| Secondary | 5,113 (73.9%) | 1,806 (26.1%) | **1.88 (1.74-2.03)** | **1.40 (1.29-1.52)** |
| Primary | 1,991 (62.9%) | 1,174 (37.1%) | **2.67 (2.46-2.89)** | **1.81 (1.66-1.98)** |
| **Personal income** |  |  |  |  |
| >8,001 EUR | 785 (87.2%) | 115 (12.8%) | 1 (ref.) | 1 (ref.) |
| 5,601–8,000 EUR | 2,133 (85.3%) | 368 (14.7%) | 1.16 (0.95-1.41) | 1.14 (0.94-1.39) |
| 4,001–5,600 EUR | 4,808 (84.3%) | 893 (15.7%) | **1.25 (1.04-1.50)** | 1.15 (0.96-1.38) |
| 2,401–4,000 EUR | 5,600 (77.5%) | 1,630 (22.5%) | **1.85 (1.55-2.20)** | **1.38 (1.15-1.65)** |
| 1,201–2,400 EUR | 3,363 (64.4%) | 1,857 (35.6%) | **3.03 (2.54-3.61)** | **1.68 (1.40-2.02)** |
| <1,200 EUR | 1,192 (74.9%) | 399 (25.1%) | **2.36 (1.94-2.87)** | **1.27 (1.04-1.56)** |
| **Employment status** |  |  |  |  |
| Active | 16,145 (81.2%) | 3,733 (18.8%) | 1 (ref.) | 1 (ref.) |
| Inactive | 1,736 (53.2%) | 1,529 (46.8%) | **2.66 (2.53-2.79)** | **2.05 (1.94-2.16)** |
| **Work Schedule** |  |  |  |  |
| Fixed | 10,841 (84.0%) | 2,070 (16.0%) | 1 (ref.) | 1 (ref.) |
| Flexible | 1,962 (79.1%) | 519 (20.9%) | **1.33 (1.22-1.45)** | **1.28 (1.18-1.40)** |
| Shift Work | 1,615 (73.5%) | 583 (26.5%) | **1.77 (1.63-1.92)** | **1.51 (1.39-1.64)** |
| Unemployed | 3,463 (62.4%) | 2,090 (37.6%) | **2.49 (2.36-2.63)** | **1.99 (1.88-2.11)** |
| **Region** |  |  |  |  |
| Reykjavík capital area | 12,144 (78.9%) | 3,239 (21.1%) | 1 (ref.) | 1 (ref.) |
| East Iceland | 462 (77.4%) | 135 (22.6%) | 1.07 (0.92-1.25) | 0.94 (0.80-1.09) |
| North Iceland | 1,797 (73.3%) | 656 (26.7%) | **1.27 (1.18-1.36)** | **1.07 (1.00-1.15)** |
| South Iceland | 1,302 (75.4%) | 424 (24.6%) | **1.16 (1.06-1.27)** | 0.98 (0.90-1.07) |
| Southern Peninsula | 794 (69.3%) | 351 (30.7%) | **1.46 (1.33-1.60)** | **1.17 (1.07-1.28)** |
| Westfjords | 260 (72.6%) | 98 (27.4%) | **1.29 (1.09-1.53)** | 1.12 (0.95-1.31) |
| West Iceland | 866 (75.1%) | 287 (24.9%) | **1.18 (1.06-1.31)** | 1.02 (0.92-1.13) |
| Living abroad | 256 (78.0%) | 72 (22.0%) | 1.06 (0.86-1.30) | 0.93 (0.76-1.13) |
| **Response period** |  |  |  |  |
| Summer (Jun-Sep) | 3,697 (80.4%) | 899 (19.6%) | 1 (ref.) | 1 (ref.) |
| Fall (Oct-Nov) | 2,464 (76.8%) | 743 (23.2%) | **1.20 (1.10-1.30)** | **1.14 (1.05-1.23)** |
| Winter (Dec-Mar) | 9,265 (75.8%) | 2,957 (24.2%) | **1.25 (1.17-1.34)** | **1.24 (1.16-1.32)** |
| Spring (Apr-May) | 2,455 (78.7%) | 663 (21.3%) | **1.09 (1.00-1.19)** | 1.07 (0.98-1.17) |
| **Body max index** |  |  |  |  |
| Normal weight | 6,794 (81.9%) | 1,506 (18.1%) | 1 (ref.) | 1 (ref.) |
| Underweight | 175 (76.4%) | 54 (23.6%) | **1.29 (1.02-1.64)** | 1.07 (0.85-1.34) |
| Overweight | 5,857 (78.6%) | 1,599 (21.4%) | **1.18 (1.11-1.26)** | **1.14 (1.07-1.21)** |
| Obesity | 5,055 (70.6%) | 2,103 (29.4%) | **1.63 (1.53-1.72)** | **1.40 (1.32-1.48)** |
| **Smoking** |  |  |  |  |
| Never | 9,284 (83.0%) | 1,898 (17.0%) | 1 (ref.) | 1 (ref.) |
| Previous | 6,290 (74.5%) | 2,156 (25.5%) | **1.51 (1.42-1.59)** | **1.35 (1.28-1.43)** |
| Non-daily | 1,011 (73.4%) | 367 (26.6%) | **1.57 (1.42-1.72)** | **1.43 (1.30-1.57)** |
| Daily | 1,296 (60.6%) | 841 (39.4%) | **2.32 (2.17-2.48)** | **1.68 (1.57-1.80)** |
| **Binge drinking in past year** |  |  |  |  |
| Never | 8,425 (77.8%) | 2,404 (22.2%) | 1 (ref.) | 1 (ref.) |
| Less than once a month | 7,117 (78.4%) | 1,962 (21.6%) | 1.00 (0.94-1.05) | **1.09 (1.04-1.15)** |
| Monthly | 1,773 (74.4%) | 610 (25.6%) | **1.18 (1.08-1.28)** | **1.27 (1.18-1.37)** |
| Once or more a week | 566 (66.4%) | 286 (33.6%) | **1.53 (1.38-1.69)** | **1.53 (1.38-1.69)** |
| **Leisure based screen time  per day** |  |  |  |  |
| < 3 hours | 8,985 (81.5%) | 2,043 (18.5%) | 1 (ref.) | 1 (ref.) |
| 3$-$5 hours | 5,143 (75.0%) | 1,713 (25.0%) | **1.37 (1.29-1.45)** | **1.22 (1.15-1.29)** |
| 5$-$7 hours | 1,929 (72.0%) | 749 (28.0%) | **1.54 (1.43-1.66)** | **1.29 (1.20-1.38)** |
| > 7 hours | 1,924 (70.7%) | 757 (29.3%) | **1.62 (1.51-1.74)** | **1.45 (1.35-1.55)** |
| **Depressive symptoms** |  |  |  |  |
| None/mild | 14,563 (88.1%) | 1,963 (11.9%) | 1 (ref.) | 1 (ref.) |
| Moderate | 2,178 (61.7%) | 1,352 (38.3%) | **3.44 (3.25-3.65)** | **3.03 (2.85-3.22)** |
| Moderately severe/   severe | 1,140 (36.9%) | 1,947 (63.1%) | **5.81 (5.53-6.11)** | **4.68 (4.43-4.94)** |
| **Anxiety symptoms** |  |  |  |  |
| None/mild | 15,287 (84.1%) | 2,887 (15.9%) | 1 (ref.) | 1 (ref.) |
| Moderate | 1,732 (58.8%) | 1,216 (41.2%) | **2.78 (2.64-2.94)** | **2.37 (2.25-2.51)** |
| Severe | 862 (42.7%) | 1,159 (57.3%) | **3.99 (3.79-4.20)** | **3.16 (2.99-3.33)** |

Note. The total *n*=23,143. 722 individuals with missing item(s) on the PSQI and 6,538 individuals with any unknown value
were removed from this analysis.
^*^ Expressed as proportions and as prevalence ratios (PRs) with 95% confidence intervals (CI). ^a^ Adjusted for age.

^b^ Adjusted for age, marital status, nr. of children, education, personal income, work schedule, region, and response period.

**
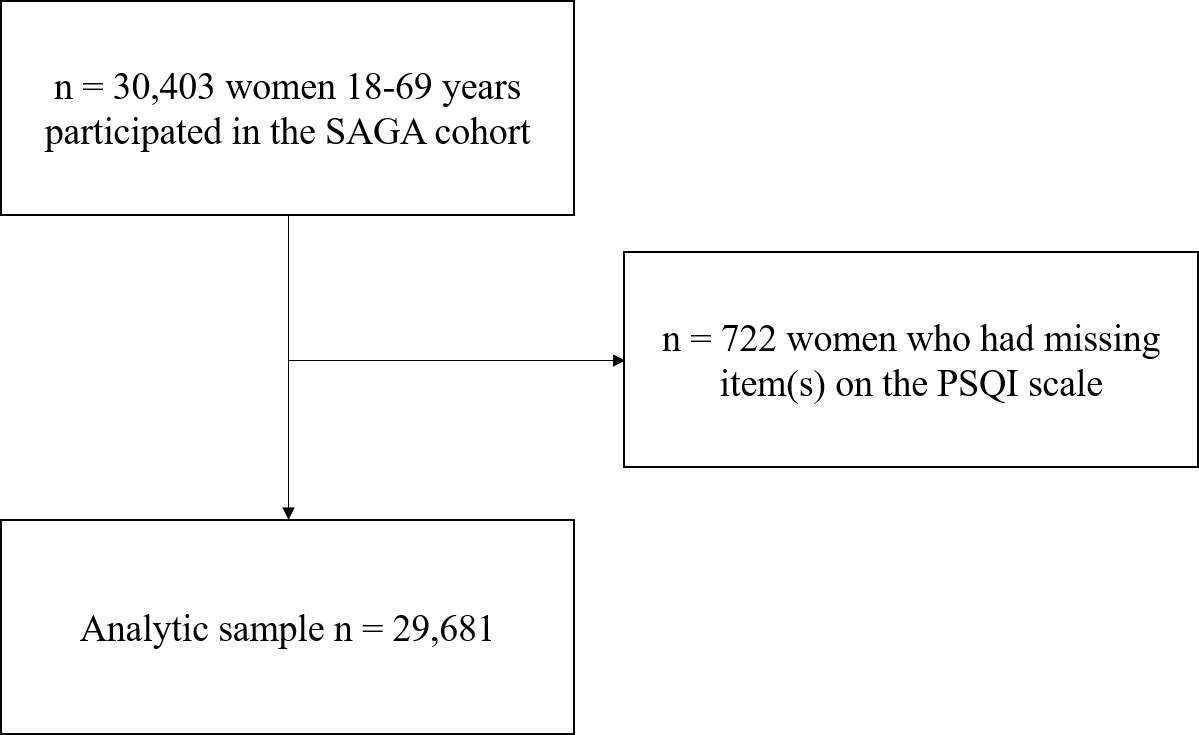
**

**Figure S1.** Flowchart of women participating in the study.


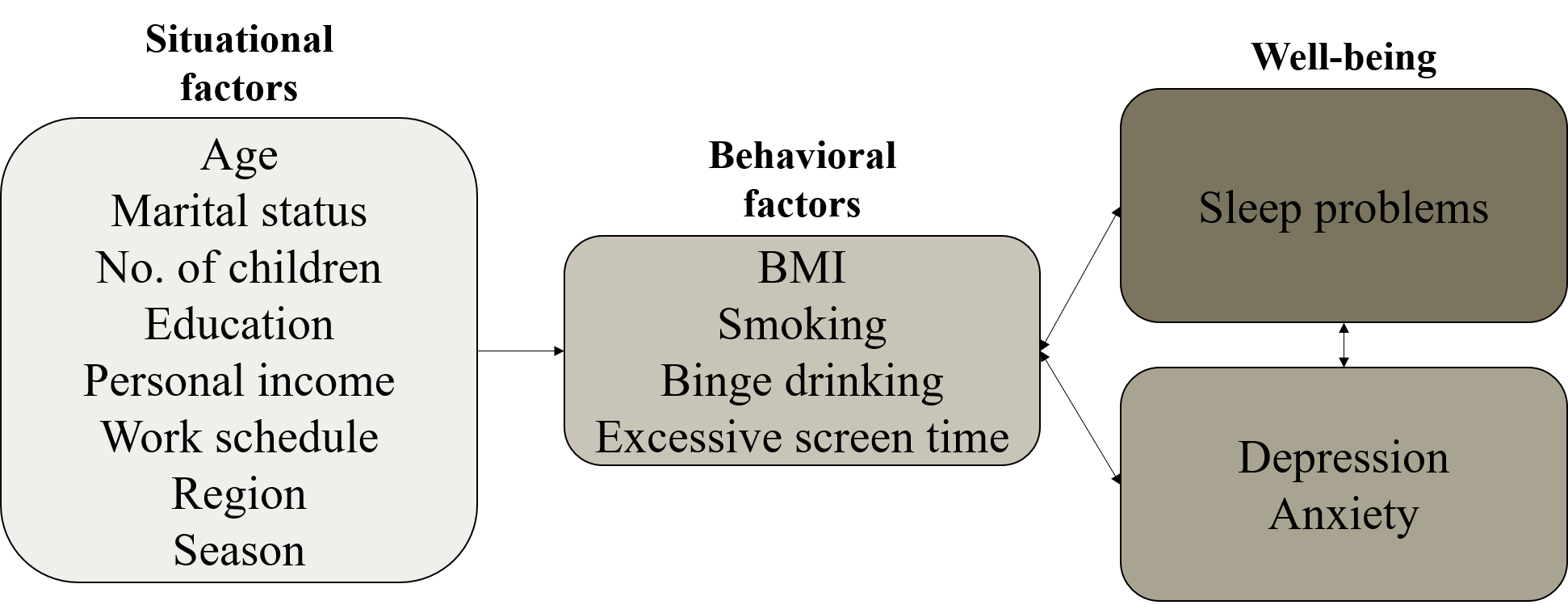


**Figure S2.** Diagram showing the relationship between variables in current study.
